# Pathophysiology and Treatments of Complications of Waldenström’s Macroglobulinemia

**DOI:** 10.46989/001c.124268

**Published:** 2024-10-07

**Authors:** Nikhil Patel, Samer Al Hadidi, Sarvari Yellapragada

**Affiliations:** 1 Internal Medicine Baylor College of Medicine; 2 Myeloma Institute University of Arkansas Medical Center; 3 Hematology Baylor College of Medicine; 4 Hematology Michael E. DeBakey VA Medical Center; 5 Hematology Dan L Duncan Comprehensive Cancer Center

**Keywords:** Waldenström’s Macroglobulinemia, Bing Neel Syndrome, Cryoglobulinemia, Hyperviscosity Syndrome, Complications

## Abstract

Waldenstrom’s macroglobulinemia (WM) or lymphoplasmacytic lymphoma is a B-cell malignancy characterized by lymphoplasmacytic cells in the bone marrow that secrete high amounts of immunoglobulin (Ig) M. The large pentameric structure of IgM leads to a variety of unique complications in WM, such as hyperviscosity syndrome, cryoglobulinemia and sensory neuropathy. Furthermore, malignant cells can infiltrate the central nervous system and lead to a variety of neurological complications, also known as Bing Neel Syndrome. Because of the unique pathophysiology of WM and these complications, their diagnostic work up and treatment regimens vary greatly. Given the rarity of the disease and their complications, there are little to no randomized controlled trials regarding treatments of these complications and, therefore, suggested treatment regimens are usually based on observational studies. In this case series, we will present three cases of WM, each with their own unique complication, and discuss the pathophysiology along with current and future treatment options for each of the complications presented.

## 1. Introduction

Waldenstrom’s macroglobulinemia (WM) is a rare hematologic malignancy that consists of lymphoplasmacytic cells (LPCs) in the bone marrow and a monoclonal immunoglobulin (Ig) M spike in the serum. The prevalence of WM is only 1,000-1,500 new cases per year in the United States and accounts for only 2% of all hematologic malignancies.^1^ Given that this is a B-cell malignancy, the LPCs in WM contain CD19 and CD20 antigens, and usually do not express CD5 and CD10 antigens. Somatic mutations in LPCs can assist in diagnosing and assessing future treatment response in WM. In 2012, whole genome sequencing of WM patients found a highly prevalent, specific mutation in chromosome 3 in a gene called MYD88, that is present in 95% of patients with WM. Another mutation, in a gene named CXCR4, is present in 40% of WM patients.^2^ Not only do these mutations help distinguish between WM and other similar hematologic malignancies like myeloma, but they are also predictive with regards to response rates to ibrutinib and other Bruton’s tyrosine kinase (BTK) inhibitors in WM. Patients with MYD88 L265P mutations responded much more favorably to ibrutinib, while those with CXCR4 mutations did not.^3^ In a study by Treon et. al, those with a MYD88 L265P mutation and wild type CXCR4 were found to have a 91.2% major response rate to ibrutinib, while in those with a MYD88 and CXCR4 mutation that level of response was 61.2%. Patients without an MYD88 mutation but with a CXCR4 mutation had the worst major response rate to ibrutinib at 28.6%.^3^ In 2016, National Comprehensive Cancer Network (NCCN) guidelines were updated to include MYD88 L265P testing in patients suspected to have WM.^4,5^ However, the current NCCN guidelines do not consider CXCR4 testing to be essential, and it should only be done in those patients being considered for BTK inhibitor therapy.

BTK is a receptor on the surface of B cells that assists with cell proliferation and immunoglobulin production. In patients with X-linked agammaglobulinemia, a mutated BTK receptor leads to B-cell immaturity and significantly decreased or absent immunoglobulin production. By using BTK inhibitors like ibrutinib in WM, this can significantly reduce the proliferation of LPCs, thereby significantly reducing IgM levels in the blood,.^6^ Newer BTK inhibitors are more selective towards BTK receptors and have a stronger bond, which has resulted in decreased side effect profiles, compared to ibrutinib and other systemic chemotherapies. Some of these newer BTK inhibitors include acalabrutinib and zanubrutinib.^7^

**Fig. 1.**
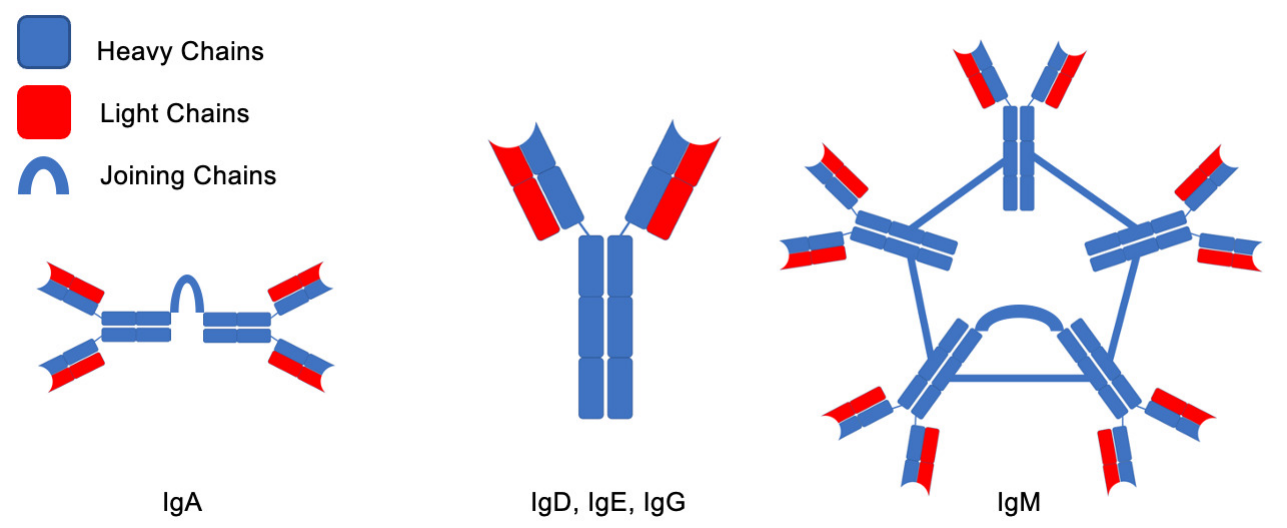
Heavy and light chain arrangements of various immunoglobulins. IgM molecules are substantially larger and bulkier compared to other immunoglobulins. The light chains labeled in red are composed of kappa and lambda units that can be measured in the serum and urine in abnormal ratios in WM^11^

Despite the rarity of the disease, there are a variety of unique complications usually present at the time of diagnosis (Table 1). The pathophysiology of these complications is mostly related to the pentameric structure of the IgM being produced by LPCs, and include amyloidosis, autoimmune hemolytic anemia, renal disease, increased risk of bleeding, neuropathy and organomegaly. The IgM molecules are unique among immunoglobulins. While other immunoglobulins have a monomeric structure consisting of light and heavy chains, IgM cross links 5 of these structures to form a large, pentameric molecule (Fig. 1). They can also precipitate in cold temperatures and lead to cryoglobulinemia, and increase the viscosity of the serum leading to hyperviscosity syndrome (HS). Furthermore, these IgM molecules can infiltrate the peripheral nervous system and lead to a variety of neurological complications, such as amyloid-induced neuropathy or anti-myelin-associated glycoprotein (anti-MAG) neuropathy.^8^ An even more rare neurological complication is Bing Neel Syndrome (BNS), which occurs when LPCs themselves infiltrate the CNS. Understanding the pathophysiology of these complications is important in indicating treatments for them along with assessing the response. In this case series, we will describe the pathophysiology of WM complications, present three cases, and discuss their treatments.

**Table 1.**
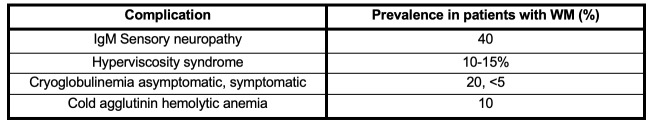
Prevalence of various complications in patients diagnosed with WM^9,10^

## 2. Case Studies

### 2.1. Hyperviscosity Syndrome

#### 2.1.1. Pathophysiology

When secreted in large enough volumes, as can be the case in WM, IgM molecules can increase the serum viscosity of blood, impede blood flow, enlarge blood vessels and lead to stasis.^12^ The viscosity is exponentially related to the amount of IgM in the blood.^13^ As such, clinical manifestations of HS include the classic triad of mucosal bleeding, visual and neurological abnormalities. Viscosity is the highest in small veins, due to low pressures and small vessel diameters. Therefore, ophthalmologic examination should be done in all patients suspected of having HS, as retinal veins are likely to be engorged in these patients in a classic “sausage-linked” pattern.^14^ Neurological signs, such as dizziness, ataxia and stroke, occur due to decreased blood flow to the CNS, along with deposition of IgM molecules in nerve sheaths. HS symptoms are found in 10 to 15% of WM patients, and are often the presenting symptom upon diagnosis.^10^ Furthermore, HS can occur after treatment of WM with rituximab, because of an IgM flare caused by the drug.^15^

#### 2.1.2. Case

In 2009, an 85-year-old male with a history of coronary artery disease and hypertension presented in the clinic with acute onset of confusion, and decreased sensation and motor function on his left leg. One month prior, he had been hospitalized for a 18-Kg weight loss and decreased sensation in his right leg. The work up had revealed a WBC count of 10,600 cells/L with 68% lymphocytes. Subsequent flow cytometry showed that 54% of cells were abnormal lambda monoclonal CD20^+^, CD5/10^-^ B cells with a serum protein electrophoresis (SPEP) showing an IgM lambda spike of 2.0 g/dL. The bone marrow biopsy showed similar CD20^+^, CD5/10^-^ B cells. The patient’s symptoms, combined with the SPEP, flow cytometry and bone marrow biopsy confirmed the suspicion for WM. Unfortunately, that hospital’s records did not include values for viscosity; however, the patient’s symptoms drastically improved with plasmapheresis, and he was diagnosed with HS in his discharge summary. He was then sent to the Veterans Affairs (VA) medical center for treatment and follow up. Given his age, less toxic chemotherapy was prioritized, and he ultimately received one cycle of dose-reduced rituximab (to prevent an IgM flare), dexamethasone, and cyclophosphamide. One day after chemotherapy administration, he was admitted for fluid overload and atrial fibrillation. Patient was discharged and then presented to the clinic one day after discharge with acute onset of confusion and decreased sensation in his left leg, and was found to have elevated serum viscosity (3.2 entipoises (cP)) with an increased serum IgM level (6.62 g/dL) compared to the previous levels two weeks prior (3.77 g/dL). The patient was diagnosed with HS given his increasing IgM levels, elevated viscosity, and new left leg motor and sensory dysfunction. After discussion with the patient, and aligned with his preferred treatment goals, it was decided that aggressive measures were not to be taken and plasmapheresis was not conducted; he was then admitted to hospice care.

#### 2.1.3. Treatment

In general, there is a variety of different ways to treat WM that depend on symptomatology, presenting complications of the disease, and severity. Usually, treatment is reserved for patients who are symptomatic. In those who present with non-acute symptoms not requiring immediate disease control, chemoimmunotherapy with bendamustine plus rituximab or ibrutinib are the most common regimens to use. Given the side effect of neuropathy with bendamustine, this is usually avoided in those whose presenting with this symptom, and they are treated with rituximab-based therapies and, possibly, ibrutinib as well. In those requiring immediate disease control, presenting with renal failure, profound cytopenia or bulky disease, chemoimmunotherapy is usually favored for immediate cytoreduction.^16^ The treatment goal of HS includes rapidly reducing IgM levels to reduce serum viscosity, increase blood flow, and subsequently reduce symptoms and organ damage. Generally, serum viscosities in HS tend to range between 4-5 cP, but can present as low as 3 cP, as was the case with our patient.^17^ The treatment algorithm for HS begins by assessing the severity of symptoms. If evidence of severe ischemia or irreversible complications are noted, empiric plasmapheresis should be conducted before laboratory results are known, and cytoreductive chemotherapy should be started promptly. Just one session of plasmapheresis can reduce IgM levels by 60% and quickly improve symptoms. In patients with mild symptoms of hyperviscosity, arguments for either aggressive cytoreductive chemoimmunotherapy or plasma exchange can be made, depending on the risk of developing HS in the future. Studies have suggested looking at serum viscosity levels in these patients to guide treatment, as high levels would indicate the need for plasma exchange, while low levels would favor chemotherapy.^14^ BTK inhibitors are able to reduce IgM levels rapidly, and may be ideal for a start immediately after plasmapheresis, in addition to cytoreductive chemotherapy such as bendamustine, rituximab, and dexamethasone, to ensure that IgM levels and LPCs are rapidly removed from the serum. Caution must be taken when starting rituximab, as its potential to increase IgM levels can worsen symptoms of hyperviscosity; therefore, it may be prudent to delay the start of rituximab.^18^

### 2.2. Bing Neel Syndrome

#### 2.2.1. Pathophysiology

Neurological complications of WM can occur due to a variety of mechanisms involving IgM and LPCs. In fact, 60% of patients with an IgM monoclonal gammopathy may develop a sensorimotor polyneuropathy.^8^ One such complication is anti-Immunoglobulin M(IgM) Anti-Myelin Associated Glycoprotein (MAG) Peripheral Neuropathy, where IgM antibodies attack glycoproteins on myelin surrounding peripheral nerves, leading to demyelination. This can manifest most commonly as a tremor, painful neuropathy, ataxia, or generalized sensorimotor dysfunction amongst other manifestations.^19^ Another neurological complication occurs when LPCs extravasate and infiltrate the CNS, in what is known as Bing Neel Syndrome (BNS). The LPCs can induce nerve demyelination, axonal degeneration, and overall CNS degradation. This is a rare complication and only occurs in up to 1% of patients with WM. Neurological dysfunction such as confusion, ataxia, and stroke-like symptoms are hallmark signs of BNS. However, these are generally non-specific, and can be confused with HS. A normal serum viscosity can help differentiate between the two. While the gold standard for diagnosis of BNS is a stereotactic brain biopsy, the less invasive approach of identifying LPCs in the CSF and MRI findings with periventricular or leptomeningeal enhancement are adequate for diagnosis.^20,21^ This leptomeningeal enhancement is generally referred to as a diffuse form of BNS, where there is diffuse enhancement within T1 weighted MRI images throughout the CNS. The tumoral form presents with discrete and focal masses within the CNS.^21^ Finally, vasogenic edema seen on MRI can be a key distinguishing factor in favor of a diagnosis of BNS over HS.^22^

#### 2.2.2. Case

**Fig. 2.**
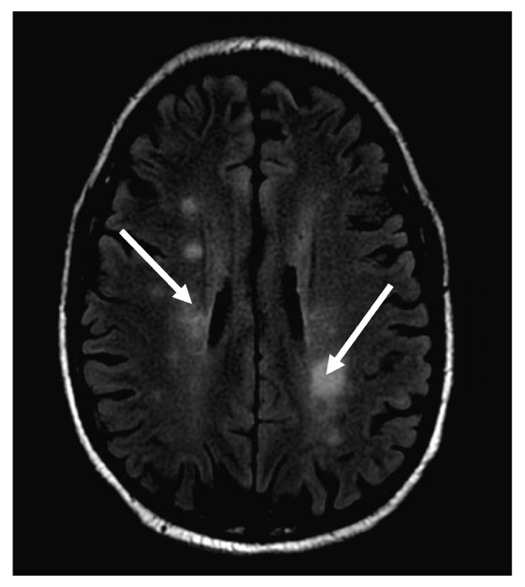
Characteristic T1-weighted MRI brain findings of Bing Neel Syndrome (BNS) where lymphoplasmacytic cells invade the CNS. The white arrows indicate areas of enhancing periventricular white matter foci that occur in BNS

In 2013, a 66-year-old male presented with a subacute onset of left-sided weakness and tremors for one month. Blood analysis revealed a WBC of 8,000 cells/micro liter with 60% atypical lymphocytes. Subsequent examination with flow cytometry, protein electrophoresis, and bone marrow biopsy showed a monoclonal B cell population secreting IgM and lymphoplasmacytic cells in the marrow that were CD 19/CD20^+^ and CD5/10^-^, consistent with WM. Serum viscosity was 1.8 (cP; ref. range 1.4-1.8 cp), indicating that these neurological symptoms were less likely caused by hyperviscosity. Although the CSF examination showed abnormal lymphocytes, it was a bloody tap, which raised the question on whether the cells were from the CSF or blood. An MRI of the brain showed indeterminate sub-centimeter enhancing white matter foci involving bilateral parietal lobes and periventricular regions, and punctate enhancing lesions in the cerebellum (Fig. 2). Given the hematologic and MRI findings, a diagnosis of BNS was made after; however the patient refused a brain biopsy to confirm the diagnosis. He was treated with whole brain radiation and dexamethasone, bortezomib and rituximab with a mild improvement in the neurological symptoms. The patient then moved to a different city, where his WM was observed for 5 years at another healthcare facility, with stable neurological symptoms and only mild increases in IgM levels. At that point, he was lost to follow up, and his death, of unknown causes, was reported one year later.

#### 2.2.3. Treatment

Treatment of neurological complications of WM vary depending on the specific complication and its mechanism. For example, antibody mediated complications like anti-MAG or amyloid neuropathy may respond to rituximab. One randomized controlled trial conducted by Dalakas et. al showed improvement in neurological symptoms in patients with anti-MAG neuropathy receiving rituximab monotherapy compared to no improvement in patients receiving placebo.^23^ Hospital et. al expanded further and conducted a retrospective study where it was found that rituximab combined with chemotherapy, such as a purine analog or cyclophosphamide, induces a faster response than rituximab monotherapy.^24^ Future directions for treatment of antibody-mediated neuropathies in WM include antibody decoys that can neutralize these antibodies within minutes and improve symptoms.^25^ Currently, treatment for BNS is usually given to symptomatic patients, and aims to halt progression of disease and neurological symptoms. There is no standardized treatment for asymptomatic patients, and it is unclear whether those with radiologic or CSF evidence of BNS should be treated. Minnema et. al stresses that CSF may still contain evidence of WM in patients who have been treated with WM, however they do not warrant additional treatment if they remain asymptomatic.^26^ Given the pathogenesis of BNS, recent studies have focused on treating these patients with CNS penetrating drugs such as nucleoside analogs and intrathecal chemotherapy; however, serious side effect profiles have been a main barrier to pursuing these treatments. In patients with only meningeal involvement on imaging, only intrathecal monotherapy may be warranted.^26^ Some of these regimens include high dose methotrexate and cytarabine, as in primary CNS lymphoma. Less toxic agents such as purine analogs and bendamustine have been tried as well.^27,28^ A literature review of 44 BNS patients showed a 70% response rate to first line treatment and a 71% five-year survival rate.^29^ Relapse can occur in up to 53% of patients with BNS, and in those who are young with aggressive disease, autologous stem cell transplant can be considered. Recommendations for conditioning therapy are based on those for primary CNS lymphoma, and favor the use of BCNU/thiotepa.^26^ New therapies, such as BTK inhibitors, with a less toxic side effect profile but good CNS penetrance, have been studied to treat BNS. A recent observational study by Castillo et al described 28 patients with BNS who received single agent ibrutinib: 85% achieved resolution of symptoms after 3 months and the five-year survival rate was 86%.^30^ Of these 28 patients, all but one had an MYD88 mutation, which, as discussed, provides a much more favorable response to ibrutinib compared to those without the characteristic mutation. None of the patients were tested for a CXCR4 mutation. In our patient, despite him not receiving any of the CNS penetrating treatments discussed above, progression of symptomatic disease was halted for at least the 5 years of follow up. Further investigations into BTK inhibitors and other CNS penetrating drugs for BNS should be conducted to help create a standardized treatment plan for this complication.

### 2.3. Cryoglobulinemia

#### 2.3.1. Pathophysiology

**Fig 3.**
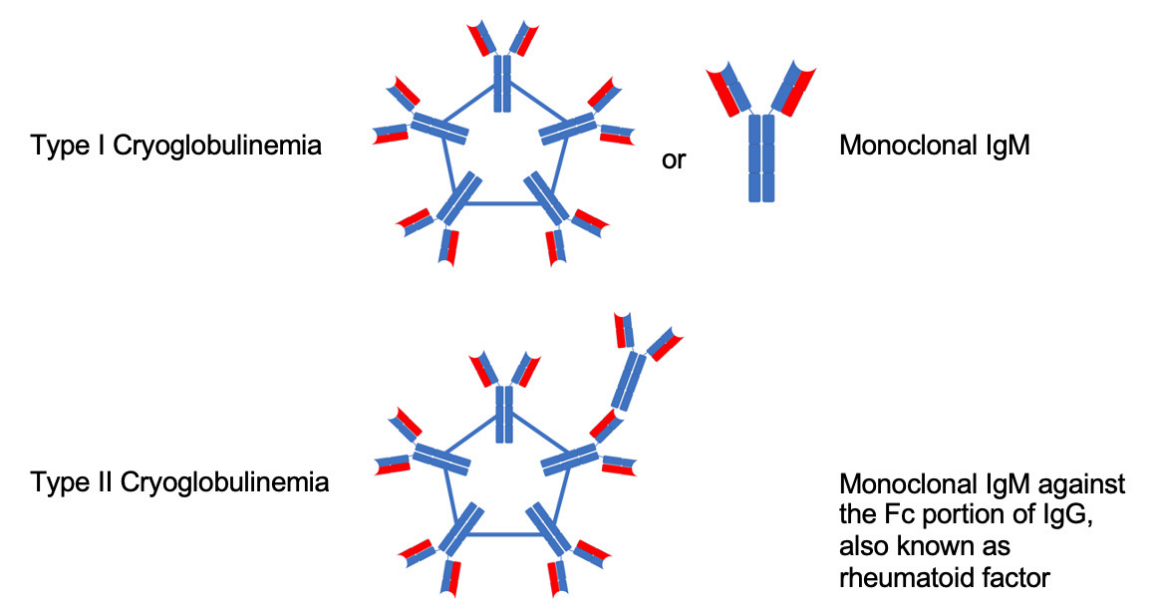
Type I and II cryoglobulinemia. In WM, Type I is mainly observed with the monoclonal IgM produced by LPCs^33^

IgM molecules can also behave uniquely at low temperatures and aggregate to form even larger molecules called cryoglobulins. Because of the increased density resulting from aggregation, cryoglobulins can precipitate out as large, solid immune complexes in the serum. Cryoglobulinemia is determined when cryoglobulins can be detected in the blood, and the cryoglobulins can precipitate into other tissues and lead to a variety of complications. In WM, we predominantly see type I cryoglobulinemia, which is characterized by cryoglobulins composed of a monoclonal Ig, usually IgM. Sometimes, WM can produce a type II cryoglobulinemia, which is usually composed of a mixture of monoclonal IgM and a rheumatoid factor (Fig. 3). Symptoms for both are similar and involve vascular complications such as ischemia, dermatologic complications such as skin necrosis and purpura, glomerulonephritis and, in type II, arthralgias, and abdominal pain.^31^ Cryoglobulins themselves are diagnosed by their visible precipitation. This can be isolated from peripheral blood samples are cooled; other methods whenever feasible, is by tissue biopsy, for example a renal biopsy demonstrating cryoglobulin precipitation.^18,32^

#### 2.3.2. Case

In 2022, a 72-year-old male with a recent COVID infection presented with 3 weeks of epistaxis, nausea and vomiting. Physical exam was noticeable for bilateral lower extremity skin changes, characterized as palpable purpura and darkening. His blood analysis showed pancytopenia, with lower than normal levels of white blood cells (WBC; 2,200 cell/microliter, ref. range 5,000-10,000 cell/microliter), hemoglobin (7.7 g/dL, ref. range 13-17 g/dL), and platelets (94,000/L, ref. range 150,000-300,000/L), and acute kidney insufficiency, as reflected by higher than normal levels for creatinine (1.7 mg/dL, baseline 1.0 mg/dL). The reticulocyte index was 0.88 (ref. range >2) indicating that his anemia was likely due to possible lack of bone marrow production. Despite fluid restoration, the kidney failure worsened. A kidney biopsy showed cryoglobulins, with evidence of glomerulonephritis. Work-up for the cryoglobulinemia included negative Hepatitis C and direct antiglobulin testing, but a positive rheumatoid factor (>650 IU/mL), indicating the patient likely had a type II cryoglobulinemia. SPEP showed a monoclonal IgM lambda spike of 0.6 g/dL and a bone marrow biopsy was conducted. The patient’s viscosity was 8.0 cP, but he did not have any symptoms of HS. Renal function continued to worsen over the next 3 days, so 2 rounds of plasma exchange and pulse dose methylprednisolone at 500mg for 3 days were given to eliminate cryoglobulins, which temporarily improved renal function. Furthermore, given the monoclonal IgM and cryoglobulinemia, there was a high suspicion of WM or another plasma cell neoplasm. Therefore, while awaiting results of the bone marrow biopsy and MYD88 testing (because of the unknown MYD88 mutation status, ibrutinib was not administered), the patient was empirically given 2 doses of bortezomib infusions, but was switched to oral ixazomib because he did not want to travel for infusions. The patient’s renal function and skin changes on his legs worsened over the next 3 months and, thus, rituximab infusions were started in conjunction with steroids and bortezomib. The bone marrow results showed a mix of CD20^+^ B-cell lineages and CD138^+^ plasma cell lineages. A repeat bone marrow biopsy was therefore conducted to help elucidate the etiology: it showed greater than 10% plasma cells, confirming WM with a negative MYD88 mutation. The patient received bortezomib with dexamethasone and has had a drastic improvement in his kidney function, IgM levels, and cryoglobulin levels with this regimen. He continues to be followed up closely every two months in the clinic, and has had fluctuating but overall stable kidney function and symptoms on his current regimen.

#### 2.3.3. Treatment

Like for BNS, treatment for cryoglobulinemia in WM should be given to patients who are symptomatic or with end organ dysfunction. The goal in the acute setting in severely symptomatic patients is to eliminate immune complexes in the blood and start cytoreductive therapy. Measurements in IgM levels and SPEP readings should be tracked to monitor treatment progress. For patients with WM and cryoglobulinemia presenting with acute end organ dysfunction, as was the case with our patient with acute renal failure, plasma exchange and steroids are warranted to rapidly eliminate immune complexes in the blood.^34^ Concurrent cytoreductive chemotherapy with bortezomib- or rituximab-based therapies should also be started, which can help reduce symptoms by up to 90%.^25,28,34^ Some of these regimens include bendamustine and rituximab, possibly along with dexamethasone.^28^ Since rituximab can cause transient increases in IgM levels, often referred to as an IgM flare, which can increase serum viscosity, IgM levels and serum viscosity levels should be measured before treatment.^34^ Given the pathophysiology of cryoglobulinemia, it would be prudent to explore the efficacy of BTK inhibitors in the acute and chronic setting (to rapidly reduce IgM levels) for its treatment in WM. There have been no randomized trials, retrospective studies or meta-analyses that have explored this topic, but some case reports have shown improvement in cryoglobulinemia symptoms and IgM levels with ibrutinib.^35^ Our patient with BNS and cryoglobulinemia did not have an MYD88 mutation that would have conferred favorability towards BTK inhibitor treatment response and, thus, was instead treated with plasmapheresis and chemotherapy.

## 3. Conclusion

Given the rarity of WM, conducting randomized controlled trials to treat its complications can be difficult due to patient recruitment. As a result, there is no standard therapy for WM. Regardless, future directions should focus on conducting combination drug trials with the newly found BTK inhibitors to find the best treatments for its complications. With current advancements in treatment, the survival of WM has drastically improved. An analysis of over 5,000 patients with WM conducted from the Surveillance, Epidemiology, and End Results database showed that the overall survival improved by 2 years from the decade after the turn of the 21^st^ century compared to the decade before. Furthermore, the current 10-year survival rate is 66%, with survival increasing in all observed age groups.^36,37^ Each complication has unique features and, as observed in these cases, will require individualized treatment regimens to improve patient outcomes and survival in the future, based on pathophysiology. The versatility of BTK inhibitors regarding their ability to penetrate the CNS and reduce IgM levels rapidly, without a strong side effect profile, makes them strong candidates for further investigation as a general treatment for many of the complications related to WM.

The future of treatments for WM and its complications extend beyond that of traditional chemoimmunotherapy. The use of stem cell transplantation for BNS has been discussed and explored, and recent developments in CAR-T cell therapies can provide future treatment options for these patients as well. An anti-20 CAR-T cell therapy for WM and other B-cell lymphomas is in Phase I/II clinical trials and has been granted orphan drug designation by the FDA.^38^ Future directions should focus on integrating chemoimmunotherapy with these new therapies to potentially increase response and survival in WM.

### Statements/Declarations

No statements or declarations to be made, there are no financial or non-financial interests. Figures made in Microsoft Word and obtained from patient charts
